# Impact of using virtual avatars in educational videos on user experience

**DOI:** 10.1038/s41598-024-56716-9

**Published:** 2024-03-19

**Authors:** Ruyuan Zhang, Qun Wu

**Affiliations:** Zhejiang Sci-Tech University, Hangzhou, 310029 Zhejiang China

**Keywords:** Virtual avatar, Knowledge popularization, Educational video, User experience, Structural equation modeling, Human behaviour, Statistics

## Abstract

Popularization of knowledge is of considerable importance and necessity, and traditional knowledge popularization activities suffer from high cost and low acceptance, which affect their effectiveness and coverage. Applying virtual avatars to educational videos may be an effective way to solve the problem. This study investigates the impact of applying virtual avatars to educational videos on user experience. Constructed a model of the impact of user experience on educational videos with virtual avatars, collected data from the target population, and analyzed it empirically. The video quality and virtual avatar expressiveness dimensions of the influencing factors have a significant positive effect on the learning effect, emotional experience and user engagement dimensions of user experience; the content quality dimension of the influencing factors has a significant negative effect on the three dimensions of user experience. The video quality and virtual avatar expressiveness dimensions of the influencing factors have a significant positive effect on the learning effect, emotional experience and user engagement dimensions of user experience; the content quality dimension of the influencing factors has a significant negative effect on the three dimensions of user experience.

## Introduction

In today's era of information explosion, a huge amount of valuable information and knowledge has been created. The popularization of science is of great social significance as it conveys such knowledge to the public and encourages them to learn and accept it. As early as 2001, the United Nations established the International Day of Science and Peace to raise public awareness of science and knowledge and to promote public participation in science popularization activities. Governments have also actively participated in international cooperation programs to promote the exchange and sharing of knowledge and popularization of science, such as the International Science Education and Scientific Knowledge Dissemination Program promoted by UNESCO. The importance and necessity of knowledge popularization can be seen from the fact that China has made the development of the country through science and education a basic national policy.

Although knowledge popularization activities have achieved certain results, there are still many problems that affect their effectiveness and coverage. The advantage of traditional knowledge popularization activities is that they are able to conduct on-site experiments and demonstrations^1^, such as optical experiments, which can visually present scientific knowledge and enhance the audience's learning experience. At the same time, traditional knowledge promotion activities can also be based on very close geographic location, to establish a combination of science, education and business knowledge popularization operation system, such as long-term cooperation system between universities, museums and communities^2^, so as to provide more in-depth science education and business experience.

However, the cost of traditional knowledge popularization activities is relatively high. Take the common exhibition format as an example, it includes exhibition design and production costs, venue rental costs, publicity and promotion costs, personnel costs, etc., and when it comes to experimental equipment and physical models, it is necessary to consider the costs of their logistics and transportation, and equipment maintenance. High costs limit traditional knowledge popularization activities, making it difficult to carry them out on a larger scale.

In addition, the limitations of knowledge dissemination are not only related to costs. Traditional knowledge popularization activities require people to be physically present, a feature that actually limits the speed of knowledge dissemination. Although many organizations publicize their offline activities through official media and other channels, traditional knowledge popularization activities in an offline location are always limited by geographic location and cannot effectively increase the speed and impact of knowledge dissemination.

On the other hand, with the advent of the information age, many institutions, organizations and individuals are making attempts to use the Internet to popularize and disseminate knowledge, such as online education platforms, official websites, online library systems and social media platforms, etc., but many people are not interested in the content and are not very receptive to it.

With the rapid development of the Internet, educational videos have become an important way for people to acquire knowledge^3,4^, and the introduction of virtual avatars in educational videos can be an effective solution to the above problems. Virtual avatar is a virtual character based on human image generated by computer technology^5^, driven by a real person or artificial intelligence, with realistic or anime style appearance and behavioral performance ability. In past studies, many scholars have demonstrated the positive effects and impacts of virtual avatars in online videos and live streaming, such as the ability to increase the attractiveness and entertainment of the videos, and to make the users more interested, engaged, and willing to pay for the videos^6,7^. Currently, virtual avatars are increasingly used in video creation, including educational videos^8–10^. By using virtual avatars, video creators hope to improve the visual appeal of videos and provide more interesting content presentation.

However, there is still a lack of systematic research on the impact of introducing virtual avatars into educational videos on user experience. Currently, there is less research on the use of avatars in educational content. Earlier studies recognized that avatars have advantages such as being able to guide users and enhance their interest in the human–computer interface^11^, and explored the psycho-emotional model of avatars in online learning software^12^. However, the limitation is that the studies examined avatars only as part of the human–computer interface, and the quantitative relationship between motivation, stimulation, and emotion was constructed only based on the e-learning software interface, which lacked the connection with the actual educational content. A portion of the research introduced avatars into e-learning videos, examining the effectiveness of high frame rate facial animation avatars and voice transducers in e-learning and comparing them with traditional live instructor videos^13,14^. The limitation is that it only investigated the attractiveness of different avatars and audio to students, and lacked a quantitative examination of the actual learning effects on students. The use of virtual avatars in educational videos has the advantages of increasing the attractiveness of the videos, expanding the video audience, and bringing higher click-through rates and playback volume. However, it should be noted that the communication center of educational videos is the content, and the purpose of communication is to improve the user's knowledge and understanding of the level of knowledge. While using virtual avatars as a way to improve the content presentation of science learning videos and stimulate users' interest in knowledge, it is also necessary to consider their potential impact on users' learning effect.

Therefore, studying the user experience influencing factors and their relationships of this type of video can provide theoretical support for the use of virtual avatars by creators of learning science videos, improve user experience and learning effect, and promote the development of science popularization activities and the dissemination of knowledge. In this study, structural equation modeling will be used to construct a model of user experience and its influencing factors for learning educational videos containing virtual avatars, and explore the relationship between multiple influencing factor dimensions and multiple user experience dimensions, and quantitatively examine the actual learning effects of users by combining them with actual educational contents.

## Research model and hypotheses

Web videos containing virtual avatars not only have common features as web videos, but also have their own unique features. Therefore, when taking popular science content as an example and constructing a research model of user experience of web videos with virtual avatars, a comprehensive model should be established by taking into account the characteristics of virtual avatars and user-generated web videos.

Video quality refers to the performance of video content in terms of clarity and smoothness. Video quality, as one of the criteria for measuring the visual effect and viewing experience of a video file or streaming media, is a key factor to focus on for educational videos containing virtual avatars. Content quality refers to the performance of video content in terms of difficulty, information content and uniqueness. Dissemination content is the core of educational videos, so content quality is also an important factor to focus on in learning science popularization online videos containing virtual avatars. Based on the above analysis, this study explores video quality, content quality and virtual avatar expressiveness together as important latent variable dimensions in modeling.

User experience is characterized by dynamism, context-dependence and subjectivity^15^, so the definition of user experience often varies somewhat across different research fields^16^. Among the definitions of user experience, the most influential is given by ISO 9241-210: all the reactions and outcomes that people have to a product, system, or service that are directed toward use or expectation of use^17^. This definition states that user experience is the mental sensations, physical sensations, and outcomes for the user when interacting with a product, system, or service. Among them, the outcome of the experience mainly consists of the user's perceptions and reactions, including emotional and physiological responses. In terms of the composition of user experience, most of the literature in the study does not explicitly follow theories such as situational experience theory^18^, user engagement theory^19^, optimal experience theory^20^ and other theories to divide the components of user experience, but rather, according to the needs of the actual research problem, targeted selection of user experience components. In related user experience studies, content, interface, interaction, and functionality dimensions are commonly used to measure the user experience of online platforms^21,22^; in recent years, more studies^23,24^ have emerged that use visual attractiveness as an independent dimension to measure the visual performance of interfaces. In addition, there are some application-specific studies^25,26^ that have established their more unique measurement dimension system according to the needs of their actual research questions. In terms of user experience model construction, most of the studies adopt multiple measurement dimensions pointing to the structure of user experience, while some studies combine their research objectives to extract the satisfaction of user needs as a separate mediating variable^27^, forming a complex model that includes mediating variables.

In this study, educational videos with virtual avatars are for users both a digital product provided by the video creator and a vehicle for viewing services provided by the platform. The psychological feeling of the user while watching the video is reflected in the outcome of the user experience, which is the user's emotional experience and further behaviors such as commenting and sharing. The results brought about by the user's viewing of the video correspond to the learning effect of the user's viewing of the video.

Based on the above analysis, the components of user experience in this study are learning effect, emotional experience and user engagement.

**Figure 1:**
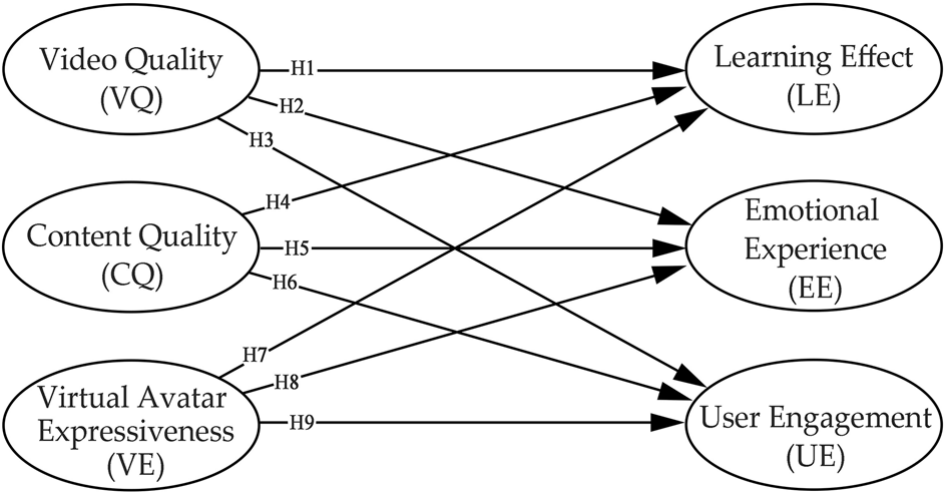
Schematic diagram of the research model of online video user experience including virtual avatar.

The first step in establishing the user experience impact model is to establish the user experience evaluation system and corresponding indicators of educational videos containing virtual avatars. The 52nd Statistical Report on China's Internet Development and other reports, together with the user profiles shown in the official data of video websites, indicate that Generation Z, born between 1995 and 2009, is the main user of video websites, and generally has a higher education level and above. Accordingly, three representative users were purposively selected for the study to conduct a qualitative study, including a primary user, an intermediate user, and an expert. Primary users were 19-year-old females in their second year of undergraduate school, with a background in Japanese language, who had moderate virtual avatar video viewing experience and very little educational video viewing experience. The intermediate user was a 25-year-old male in the second year of his master's degree, with a background in apparel and product design, who had moderate virtual avatar video viewing experience and little educational video viewing experience. The expert is a 20-year-old male in his third year of undergraduate school, with a background in digital media arts, and has very much virtual avatar video viewing experience with moderate educational video viewing experience. The user interviews were conducted through a semi-structured interview format, with questions on viewing motives, viewing goals, and viewing scenarios for watching virtual avatar videos, educational videos, and educational videos containing virtual avatars, and follow-up questions on influencing factors related to the viewing experience. The original records of the interviews were organized and analyzed, and the latent variable factors affecting the user experience of educational videos containing virtual avatars were initially summarized, and the corresponding observational indexes were clarified to form a Likert scale. After the scale was established, in order to determine the appropriate factor structure with correcting problems in the model, a small-scale pre-experimental method was used to test it, and 68 valid data were recovered. The results of the pre-experiment showed that the discriminant validity between some of the latent variable dimensions was poor and required further modification. The modified conceptual model of this paper is shown in Fig. 1.

Video quality refers to the performance of video content in terms of clarity and smoothness. Video quality, as one of the standards for measuring the visual effect and viewing experience of a video file or streaming media, is a key factor that all types of online videos need to focus on. Video quality in this paper includes picture clarity and smoothness, sound and audio quality, video scene and screen layout, and the use of special effects. In the process of watching videos, clear and smooth audio and video playback is an important basis for users to successfully access the content. The phenomena of blurring, lagging, and unsynchronized audio and video in the video viewing process will hinder the delivery of the content, affect the user's access to the video content, trigger the generation of negative emotions^28^, and significantly reduce the user's willingness to continue to watch. With the advancement of camera, codec, bandwidth and storage technologies, as well as the popularization of high-performance displays, users have put forward higher requirements for video clarity and other attributes. Simultaneously, video creators' long-term exploration of video composition, special effects implantation and other details have further increased users' demands on the aesthetic aspects of video quality.

For video platforms, video quality is related to the social reputation and image of the platform. Higher video quality helps platforms create their own good image of network video platforms, which has a direct impact on the platform traffic; at the same time, generally high-quality videos can help to stimulate a positive emotional experience for users, which in turn improves their loyalty to the video platform. For video creators, video quality is the embodiment of their own creative level and ability, but also the basis for video creators to accumulate the number of fans and improve fan stickiness. For individual users, video quality not only affects the viewing decision when facing similar videos, but also is closely related to the user experience when and after watching the video. Based on the above analysis, this paper proposes the following hypotheses:

- H1: Video quality has a positive effect on learning effect;
- H2: Video quality has a positive effect on emotional experience;
- H3: Video quality has a positive effect on user engagement.

Content quality refers to the performance of video content in terms of difficulty, information and uniqueness. The communication content is the core of learning science educational videos, so content quality is an important factor to focus on for learning science online videos containing virtual avatars. Bloom's taxonomy of cognitive goals, proposed in 1956, categorizes cognitive goals into six levels based on their complexity: memory, comprehension, application, analysis, synthesis, and creation^29,30^. Among these, memorization and comprehension correspond to lower content quality and cognitive complexity, application and analysis correspond to moderate content quality and cognitive complexity, and synthesis and creation correspond to higher content quality and cognitive complexity. In this paper, content quality is further refined into knowledge communication accuracy, content depth, content information sufficiency, content hierarchy clarity, and content uniqueness. Among them, the accuracy of knowledge conveyance refers to whether the video's representation of its content's knowledge points, definitions, and proper nouns is sufficiently accurate and rigorous. The depth of the content is the degree of specialization of the content. Usually, as the depth of the content increases, so does the degree of specialization and learning difficulty. Content information sufficiency is directly proportional to the amount of information and details contained in the video content, and is also a key factor in determining the total length of the video. The clarity of the content hierarchy refers to whether the video content shows the structure of the specific knowledge is clear, whether it is more clear to show the level of knowledge, relevance and so on. The uniqueness of the content refers to the degree of novelty and originality of the knowledge and other content disseminated by the video.

For the learning effect, under the premise of excluding videos involving erroneous content and misinformation, as the depth of the content increases, the amount of information increases, and the complexity of the knowledge structure increases, the learning difficulty of the user increases; in other words, the user needs to put in more brain power, can remember less information, and is more likely to reduce their interest in the video content. Similarly, for emotional experience, increased learning difficulty is more likely to cause frustration on the one hand, resulting in users being less prone to positive emotional tendencies. Additionally, the number of users successfully digesting the video content decreases, which in turn decreases the rate of positive emotions such as satisfaction and fulfillment. User engagement is also affected by content quality to a certain extent; videos whose content difficulty matches the learning level of users tend to have higher average viewing time and completion rate, and are more likely to cause users to comment, share, and other behaviors of an engaging nature. Based on the above analysis, this paper proposes the following hypothesis:

- H4: Content quality has a negative effect on learning effect;
- H5: Content quality has a negative effect on emotional experience;
- H6: Content quality has a negative effect on user engagement.

While virtual avatars are introduced as technical tools for video creation, they are also used as visual elements that are part of the video screen. Virtual avatar expressiveness refers to the overall visual performance and attractiveness of the virtual avatars. In this paper, virtual avatar expressiveness includes visual attractiveness, emotional expressiveness, naturalness of movement, and naturalness of look.

Virtual avatars are inherently entertaining and are able to portray themselves through a rich variety of appearance features, clothing styles, and animation effects, and this visual attraction can stimulate users' curiosity and appreciation, resulting in a positive emotional experience. At the same time, this visual attraction can strengthen users' interest in the video^11,13,14^, increase concentration, and make them more willing to invest time and energy^31^. The virtual avatar interacts with users through voice and verbal expression in the video, and its unique voice and tone, which expresses a variety of emotions, attitudes and moods and establishes its own personality, on the one hand, it can promote the establishment of a more intimate connection between the user and the virtual avatar, and it can enhance the user's sense of enjoyment^7^, participation^32^ and emotional resonance, so that the user is more willing to continue to watch and interact with the video^4^; On the other hand, this intuitive verbal communication can enhance users' acceptance and digestion of knowledge, and promote the learning effect^33^. The naturalness of movement and the naturalness of expression reflect the degree of natural and smooth movement and the degree of exquisite and delicate expression of the virtual avatar, respectively. By displaying a variety of dynamic gestures and expressions, emotions such as joy, anger, sadness and happiness are conveyed through body language. This vivid form of expression allows users to more intuitively feel the information^34^ and emotions conveyed by the virtual avatar^35^, thus deepening their emotional recognition and affection for the virtual avatar, encouraging them to leave comments, share them with other people, pay attention to the subsequent updates, and deepen their impression of the video, thereby consolidating their knowledge learning. Based on the above analysis, this paper proposes the following hypotheses:

- H7: Virtual avatar expressiveness has a positive effect on learning effect;
- H8: Virtual avatar expressiveness has a positive effect on emotional experience;
- H9: Virtual avatar expressiveness has a positive effect on user engagement.

The pointing relationship of all hypotheses is shown in Fig. 1.

## Methods

This study is based on a questionnaire to collect data. The design of the research questionnaire was based on mature literature and scales at home and abroad, and it was revised for the research object of this study to make the questionnaire more in line with the research needs of this paper. The questionnaire is based on a 5-point Likert scale, with 1 point indicating disagreement and 5 points indicating agreement. The specific questions of the research questionnaire are as follows.

Video quality and content quality. Video quality and content quality are aspects that need to be considered for all types of videos. The video quality can directly affect the user's perception and experience of the video. For example, a high-definition video will make viewers enjoy and continue to watch the video more. Based on existing research, this paper uses picture clarity and smoothness, sound effects audio quality, video scene and screen layout and special effects use to measure overall video quality. Good content quality can reflect the educational value and appeal of a video while improving the overall user experience. For example, videos with accurate knowledge conveyance can increase users' trust and satisfaction with the content. Based on existing research, this paper uses knowledge communication accuracy, content depth, content information sufficiency, content hierarchy, and content uniqueness to measure overall content quality.

Virtual avatar expressiveness. Virtual avatar expressiveness is an important source of video's attractiveness to users, and this paper further divides virtual avatar expressiveness into visual attractiveness^36^, emotional expressiveness^7,35^, naturalness of movement and naturalness of look.

Learning effect. Because the educational videos containing virtual avatars have the purpose of knowledge dissemination in themselves, this paper examines the learning effect of users after watching the videos as one of the latent variables of the overall user experience. In this paper, the learning effect is further classified into knowledge transmission effect, learning interest degree, learning difficulty adaptation degree and educational goal realization degree.

Emotional experience. Emotional experience refers to the user's emotional response that occurs during or after watching a video, and this emotional response directly affects the user's attitude toward a specific object, which in turn affects the user experience and decision-making^23^. Based on this, this paper examines emotional experience as a latent variable of overall user experience. Combined with existing studies^35^, this paper measures user emotional experience through user emotional tendency, value perception, emotional expression in content, and favoritism and identification.

User engagement. User engagement refers to the interactive behavior of users in the process of watching videos or after watching, and this interactive behavior can reflect the user's interest in the video, sense of identity and acceptance. Based on existing studies^37–39^, this paper further refines user engagement into three observation indicators: average viewing duration, sharing rate and social media interaction, as well as the number of comments and activity.

The latent variables of experimental interest and the corresponding observables are shown in Table 1.

**Table 1:** Indicator system of factors influencing user experience of educational videos containing virtual avatars.

Latent variable	Observed indicators
Video quality	Picture clarity and smoothness
	Audio quality
	Video Scene and Screen Layout
	Use of special effects
	Video Color Matching
Content quality	Accuracy of Knowledge Conveyance
	Depth and difficulty of content
	Sufficiency of content information
	Content hierarchy
	Content uniqueness
Virtual avatar expressiveness	Visual appeal
	Emotional expression
	Naturalness of movement
	Naturalness of expression
Learning effect	Effectiveness of knowledge transfer
	Interest in Learning
	Adaptability to Learning Difficulty
	Achievement of Educational Goals
Emotional experience	Emotional tendency of users
	Value perception
	Emotional expression in content
	Favoritism and identification
User engagement	Average viewing time
	Sharing rate and social media interaction
	Number of comments and activity

Data were analyzed using the software SPSS 26 with Amos 26.

All experiments were performed in accordance with relevant named guidelines and regulations.

Informed consent was obtained from all participants.

### Institutional review board statement

The study was conducted in accordance with the Declaration of Helsinki, and approved by the Institutional Review Board of School of Art and Design of Zhejiang Sci-Tech University (protocol code 1016/2023 and 10.16.2023).

### Informed consent statement

Informed consent was obtained from all subjects involved in the study.

## Results

### Sample feature distribution description

In this study, a total of 194 questionnaires were collected, and the research target was mainly students in colleges and universities who have experience in watching educational videos containing virtual avatars. Students in colleges and universities are not only the key target of knowledge popularization, but also the representatives of strong learning ability and fast acceptance of new knowledge. After screening, 113 valid questionnaires were obtained. Table 2 describes the distribution of sample characteristics. Among them, 65% of the research subjects were aged 18–22, representing undergraduate students, 42% were aged 23–27, representing master's level students, and a total of about 95% were aged 18–27, with a gender ratio close to 1:1, which is in line with the demand for the selection of research subjects.

**Table 2:** Description of the distribution of sample characteristics.

Variables	Options	Frequency	Percentage
Age	Under 18	0	0
	18–22	65	57.5
	23–27	42	37.1
	28 and above	6	5.3
Sex	Female	62	54.9
	Male	51	45.1

### Reliability analysis

In this study, the main factors were measured in the form of scales, so testing the data quality of the measurement results is an important prerequisite to ensure the significance of the subsequent analysis. The internal consistency of each dimension was first analyzed through the Cronbach factor reliability test. The Cronbach factor value ranges from 0 to 1, with higher values indicating greater reliability. A reliability coefficient of 0.6 or less is generally considered unreliable and requires a redesign of the questionnaire or an attempt to re-collect the data and analyze it again. A reliability coefficient between 0.6 and 0.7 is barely credible, between 0.7 and 0.8 is relatively credible, between 0.8 and 0.9 is very credible, and between 0.9 and 1 is highly credible^40^.

In this analysis, the results of the reliability analysis are shown in Table 3, where the reliability coefficients of the influencing factors and user experience in general as well as for each of the secondary dimensions are in the range of 0.7–1, indicating good internal consistency and reliability of the scales used in this study.

**Table 3:** Reliability analysis of user experience scale for educational videos containing virtual avatars.

Variant	Cronbach factor	Number of terms
Video quality	0.841	5
Content quality	0.866	5
Virtual avatar expressiveness	0.804	4
Influencing factors	0.905	14
Learning effect	0.871	4
Emotional experience	0.802	4
User engagement	0.786	3
User experience	0.913	11

### Validity analysis

#### Impact factor scale validation factor analysis

According to the results of the model fit test in Table 4, it can be seen that CMIN/DF (cardinal degrees of freedom ratio) = 1.975, which is in the excellent range of 1–3, and RMSEA (root mean square of error) = 0.093, which is in the good range of < 0.10. In addition, the test results of IFI and CFI reached an excellent level of 0.9 or more, and the test results of TLI reached a good level of 0.8 or more. Therefore, synthesizing the results of this analysis can indicate that the Impact Factor Scale CFA model has a good fit.

**Table 4:** Impact factor scale CFA model fit test.

Indicator	Reference standard	Measured result
CMIN/DF	1–3 is excellent, 3–5 is good	1.975
RMSEA	< 0.05 is excellent, < 0.10 is good	0.093
IFI	> 0.9 is excellent, > 0.8 is good	0.907
TLI	> 0.9 is excellent, > 0.8 is good	0.883
CFI	> 0.9 is excellent, > 0.8 is good	0.905

Under the precondition that the CFA model of the impact factor scale has good fit, the convergent validity (AVE) and combinatorial reliability (CR) of the dimensions of the scale will be further tested. The test procedure is to calculate the standardized factor loadings of each measurement question item on the corresponding dimension through the established CFA model. Then, the convergent validity values and combined reliability values of each dimension were calculated by the formulae of AVE and CR. According to the criteria, a minimum AVE value of 0.5 and a minimum CR value of 0.7 are required to indicate good convergent validity and combinatorial reliability.

According to the results of the analysis in Table 5, it can be seen that in this validity test of the impact factor scale, the AVE value of each dimension reached more than 0.5 and the CR value reached more than 0.7, and the synthesis can indicate that all dimensions have good convergent validity and combinatorial reliability.

**Table 5:** Convergent validity and combined reliability tests for each dimension of the impact factor scale.

Path relation	Estimate	AVE	CR
VQ1 ← Video quality	0.54	0.502212	0.831292
VQ2 ← Video quality	0.637		
VQ3 ← Video quality	0.687		
VQ4 ← Video quality	0.809		
VQ5 ← Video quality	0.829		
CQ1 ← Content quality	0.74	0.56321	0.8648261
CQ2 ← Content quality	0.731		
CQ3 ← Content quality	0.792		
CQ4 ← Content quality	0.835		
CQ5 ← Content quality	0.64		
VE1 ← Virtual avatar expressiveness	0.535	0.571361	0.8368909
VE2 ← Virtual avatar expressiveness	0.691		
VE3 ← Virtual avatar expressiveness	0.933		
VE4 ← Virtual avatar expressiveness	0.807		

**Figure 2:**
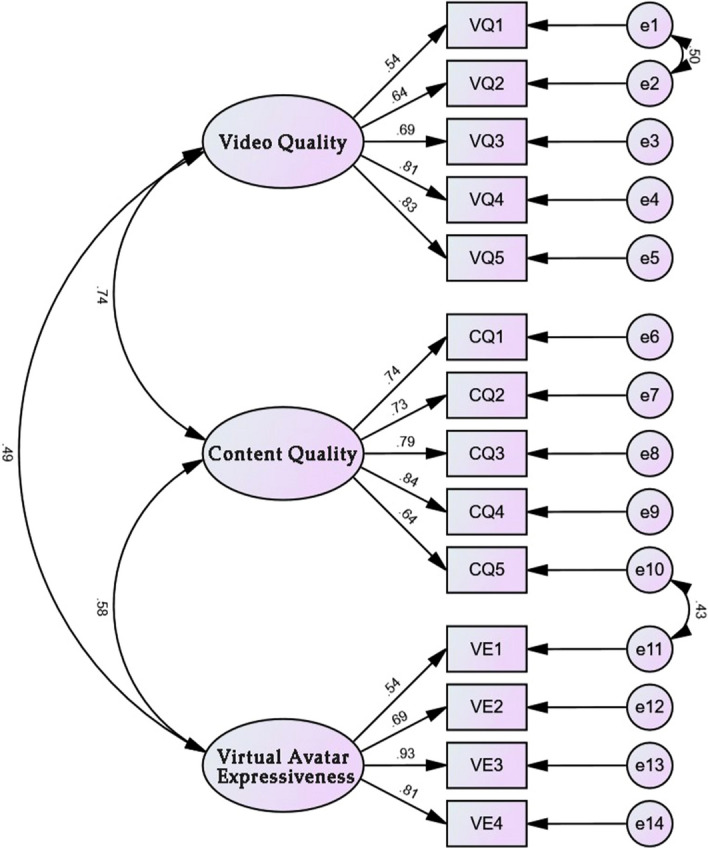
Impact factor scale validated factor analysis CFA model diagram.

According to the analysis results in Table 6, it can be seen that the standardized coefficients between the virtual avatar expressiveness dimension and the other two dimensions in this test of discriminant validity are less than the square root of the corresponding AVE values, indicating good discriminant validity between the virtual avatar expressiveness dimension and the other two dimensions. The standardized coefficients between the video quality dimension and the content quality dimension are slightly larger than the square root of the corresponding AVE values, indicating average discriminant validity between the video quality dimension and the content quality dimension. The impact factor scale validated factor analysis CFA model diagram is shown in Fig. 2.

**Table 6:** Results of distinctive validity tests for dimensions of the impact factor scale.

Variant	Video quality	Content quality	Virtual avatar expressiveness
Video quality	0.502		
Content quality	0.742	0.563	
Virtual avatar expressiveness	0.490	0.583	0.571
Square root of AVE value	0.709	0.750	0.756

#### User experience scale validation factor analysis

According to the model fitness test results in Table 7, it can be seen that CMIN/DF = 1.587, which is in the excellent range of 1–3, and RMSEA = 0.072, which is in the good range of < 0.10. In addition, the test results of IFI, TLI and CFI all reached an excellent level of 0.9 or more. Therefore, synthesizing the results of this analysis can indicate that the user experience scale CFA model has a good fit.

**Table 7:** User experience scale CFA model fit test.

Indicator	Reference standard	Measured result
CMIN/DF	1–3 is excellent, 3–5 is good	1.587
RMSEA	< 0.05 is excellent, < 0.10 is good	0.072
IFI	> 0.9 is excellent, > 0.8 is good	0.966
TLI	> 0.9 is excellent, > 0.8 is good	0.954
CFI	> 0.9 is excellent, > 0.8 is good	0.965

Under the precondition that the CFA model of the user experience scale has a good fit, the convergent validity (AVE) and combinatorial reliability (CR) of the dimensions of the scale will be further examined. The testing process is consistent with the previous section, and the standardized factor loadings of each measurement question item on the corresponding dimension are calculated through the established CFA model. Then the convergent validity values and combined reliability values of each dimension were calculated by the formulas of AVE and CR. According to the criteria, a minimum AVE value of 0.5 and a minimum CR value of 0.7 are required to indicate good convergent validity and combinatorial reliability.

According to the analysis results in Table 8, it can be seen that in this validity test of the user experience scale, the AVE value of each dimension reaches more than 0.5, and the CR value reaches more than 0.7, which comprehensively can indicate that each dimension has good convergent validity and combined reliability.

**Table 8:** Convergent validity and combined reliability test for each dimension of the user experience scale.

Path relation	Estimate	AVE	CR
LE1 ← Learning effect	0.763	0.6287193	0.871169
LE2 ← Learning effect	0.844		
LE3 ← Learning effect	0.766		
LE4 ← Learning effect	0.796		
EE1 ← Emotional experience	0.772	0.5131465	0.8077011
EE2 ← Emotional experience	0.733		
EE3 ← Emotional experience	0.652		
EE4 ← Emotional experience	0.703		
UE1 ← User engagement	0.962	0.5593787	0.7830064
UE2 ← User engagement	0.666		
UE3 ← User engagement	0.556		

**Figure 3:**
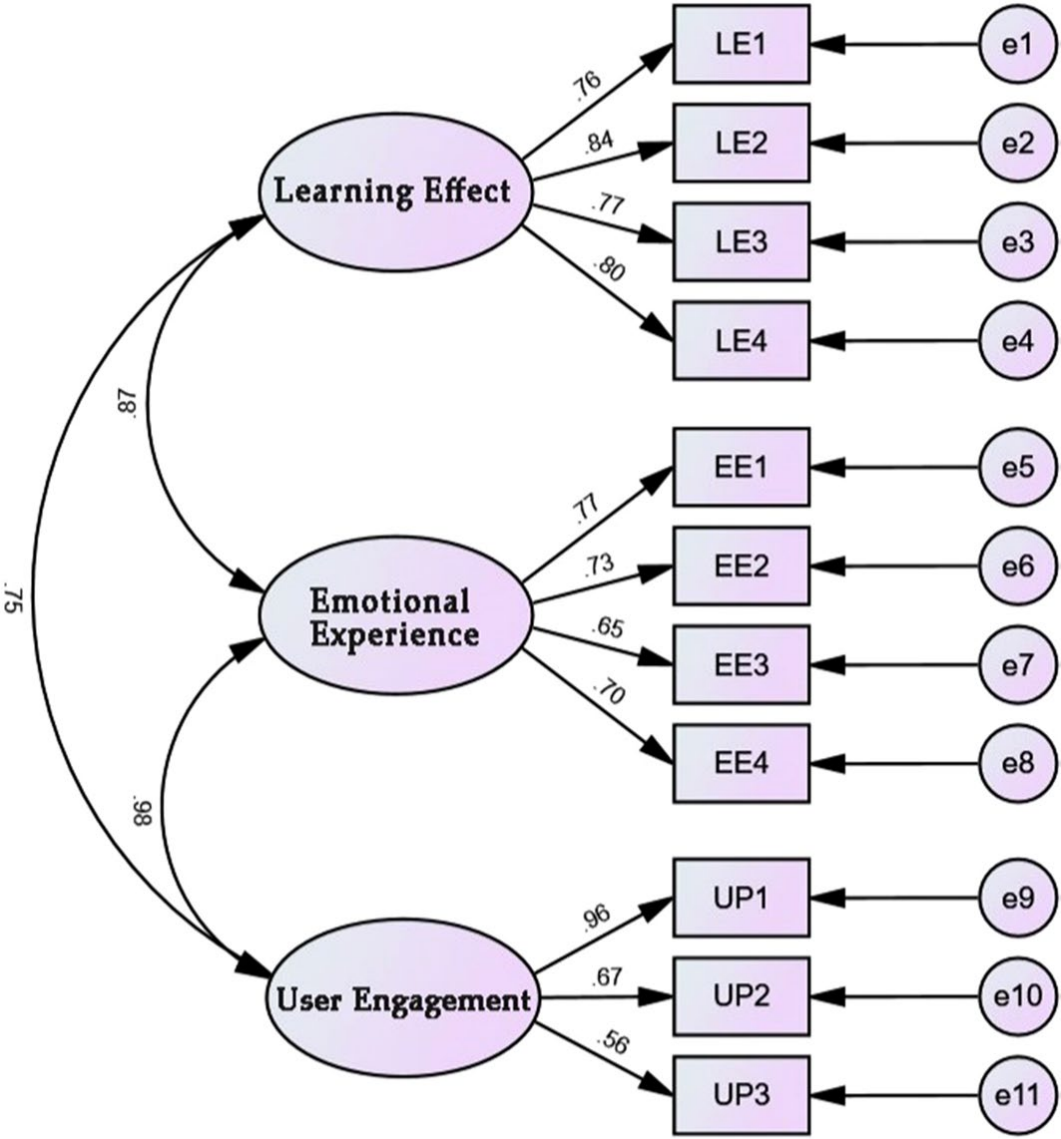
User experience scale validated factor analysis CFA model diagram.

According to the analysis results in Table 9, it can be seen that in this test of differential validity, the standardized coefficient between the learning effect dimension and the user engagement dimension is less than the square root of the corresponding AVE value, indicating that there is good differential validity between the learning effect dimension and the user engagement dimension. The standardized coefficient between the emotional experience dimension and the other two dimensions is slightly larger than the square root of the corresponding AVE value, indicating average discriminant validity between the emotional experience dimension and the other two dimensions. The user experience scale validated factor analysis CFA model diagram is shown in Fig. 3.

**Table 9:** Results of the differentiated validity test for each dimension of the user experience scale.

Variant	Learning effect	Emotional experience	User engagement
Learning effect	0.629		
Emotional experience	0.873	0.513	
User engagement	0.751	0.979	0.559
Square root of AVE value	0.793	0.716	0.748

### Descriptive statistics analysis and normality test

Table 10 below shows the results of the descriptive statistics analysis and normality test of the current status of the factors used in this study. According to the results of the descriptive statistics analysis, it can be seen that the mean scores of each variable are between 3 and 5, and the scale scoring method is 1–5 positive scoring, so it can be seen that the level of the subject group's awareness of educational videos containing virtual avatars and the level of subjective user experience are above the medium level.

**Table 10:** Results of normality test for descriptive statistics and measurement question items for each dimension.

Dimension	Items	Mean	Standard deviation (SD)	Skewness	Kurtosis	Overall mean	Overall SD
Video quality	VQ1	4.27	0.793	− 0.971	0.558	4.049558	0.684278
	VQ2	4.19	0.789	− 0.695	− 0.086		
	VQ3	3.9	0.855	− 0.247	− 0.763		
	VQ4	3.91	0.996	− 0.703	− 0.022		
	VQ5	3.96	0.925	− 0.618	− 0.103		
Content quality	CQ1	3.82	0.928	− 0.32	− 0.773	3.893805	0.724111
	CQ2	3.88	0.863	− 0.452	− 0.377		
	CQ3	3.84	0.841	− 0.241	− 0.594		
	CQ4	3.92	0.898	− 0.444	− 0.584		
	CQ5	4	0.954	− 0.69	− 0.152		
Virtual avatar expressiveness	VE1	4.03	0.881	− 0.611	− 0.337	3.7035	0.85731
	VE2	3.74	1.076	− 0.653	− 0.17		
	VE3	3.59	1.058	− 0.456	− 0.344		
	VE4	3.45	1.142	− 0.336	− 0.547		
Learning effect	LE1	3.97	0.85	− 0.748	0.659	3.9757	0.725
	LE2	4.01	0.871	− 0.595	0.099		
	LE3	3.83	0.844	− 0.487	0.252		
	LE4	4.09	0.851	− 0.879	0.856		
Emotional experience	EE1	4.03	0.85	− 0.584	0.181	3.7456	0.76618
	EE2	3.9	0.906	− 0.392	− 0.687		
	EE3	3.55	0.964	− 0.323	− 0.09		
	EE4	3.5	1.127	− 0.297	− 0.811		
User engagement	UE1	3.87	0.968	− 0.57	− 0.064	3.684366	0.858941
	UE2	3.47	1.181	− 0.488	− 0.624		
	UE3	3.72	0.911	− 0.559	0.255		

The normality test for each measurement question item was performed using skewness and kurtosis, and according to the criteria proposed by Kline it is considered that the data satisfy the requirements of approximate normal distribution if the absolute value of the skewness coefficient is within 3 and the absolute value of the kurtosis coefficient is within 8^41^. Based on the analysis results in Table 4, it can be seen that the absolute values of skewness and kurtosis coefficients for each measurement question item in this study are within the standard range. Therefore, it can be stated that the data of each measurement question item satisfy the approximate normal distribution.

### Pearson correlation analysis

In this analysis, the correlation between each variable was explored through Pearson correlation analysis. Based on the results of the Pearson correlation analysis in Table 11, it can be seen that there is a significant correlation between each variable in this analysis and all of them are significant at the 99% level of significance.

**Table 11:** Results of Pearson correlation analysis between dimensions.

Dimension	VQ	CQ	VE	LE	EE	UE
VQ	1					
CQ	.659**	1				
VE	.441**	.532**	1			
LE	.587**	.523**	.522**	1		
EE	.476**	.444**	.494**	.715**	1	
UE	.390**	.278**	.360**	.585**	.754**	1

**Significant correlation at the 0.01 level (two-tailed).

### Structural equation model

#### SEM model fit test for user experience impact factors

Based on the results of the model fit test in Table 12, it can be seen that CMIN/DF = 2.029, which is in the excellent range of 1–3, and RMSEA = 0.096, which is in the good range of < 0.10. In addition, the test results of IFI, TLI and CFI all reached a good level of 0.8 or more. Therefore, synthesizing the results of this analysis can indicate that the SEM model of user experience influencing factors has a good fit.

**Table 12:** SEM Model fitness test.

Indicator	Reference standard	Measured result
CMIN/DF	1–3 is excellent, 3–5 is good	2.029
RMSEA	< 0.05 is excellent, < 0.10 is good	0.096
IFI	> 0.9 is excellent, > 0.8 is good	0.845
TLI	> 0.9 is excellent, > 0.8 is good	0.818
CFI	> 0.9 is excellent, > 0.8 is good	0.841

#### User experience impact factors SEM model path relationship hypothesis testing results

**Figure 4:**
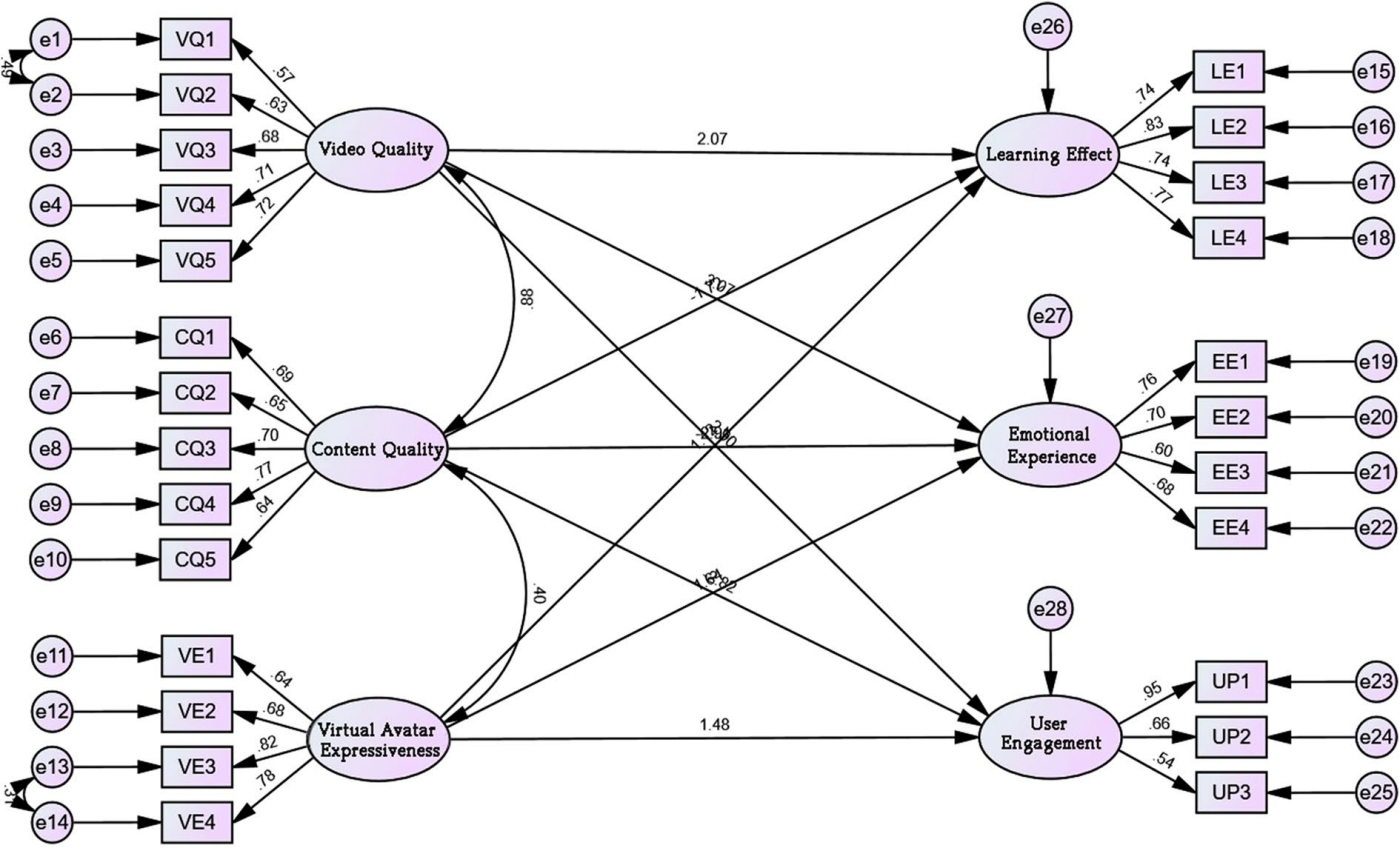
SEM analysis model diagram of user experience impact factors.

Based on the analysis results in Table 13, it can be seen that video quality significantly and positively predicts learning effect (β = 2.066, *p* < 0.001) in the path hypothesized relationship test of the current study, so hypothesis H1 is valid. Video quality significantly and positively predicts emotional experience (β = 3.067, *p* < 0.001), therefore hypothesis H2 is valid. Video quality significantly positively predicted user engagement (β = 2.899, *p* < 0.001), therefore hypothesis H3 holds. Content quality significantly inversely predicted learning effect (β = -1.723, *p* = 0.009), therefore hypothesis H4 holds. Content quality significantly inversely predicts emotional experience (β = − 2.907, *p* = 0.002), therefore hypothesis H5 holds. Content quality significantly inversely predicted learning effect (β = − 2.822, *p* = 0.002), therefore hypothesis H6 holds. Virtual avatar expressiveness significant positive predictive learning effect (β = 1.118, *p* < 0.001), therefore hypothesis H7 holds. Virtual avatar expressiveness significantly and positively predicts emotional experience (β = 1.64, *p* < 0.001), therefore hypothesis H8 holds. Virtual avatar expressiveness significantly and positively predicts user engagement (β = 1.483, *p* < 0.001), therefore hypothesis H9 holds. The SEM analysis model of user experience impact factors are shown in Fig. 4.

**Table 13:** User experience influencing factors SEM path relationship test results.

Path relation	Estimate	S.E	C.R	*P*
Learning effect ← video quality	2.066	0.554	3.396	***
Emotional experience ← video quality	3.067	0.806	3.539	***
User engagement ← video quality	2.899	1.064	3.611	***
Learning effect ← content quality	− 1.723	0.682	− 2.614	0.009
Emotional experience ← content quality	− 2.907	1.017	− 3.024	0.002
User engagement ← content quality	− 2.822	1.357	− 3.133	0.002
Learning effect ← virtual avatar expressiveness	1.118	0.211	3.575	***
Emotional experience ← virtual avatar expressiveness	1.64	0.305	3.704	***
User engagement ← virtual avatar expressiveness	1.483	0.4	3.64	***

***P value less than 0.001.

## Discussion

Compared to prior studies, this study systematically examined the impact of introducing virtual avatars into educational videos on user experience. This study innovatively quantitatively examined the effects on user learning effect, emotional experience, and user engagement by using overall virtual avatar expressiveness, video quality, and content quality as latent variables. On the one hand, it avoids the limitation of examining virtual avatars only as a part of the human–computer interface, which lacks the connection with the actual educational content; on the other hand, it also emphasizes the quantitative examination of the learning effect on the users and avoids the limitation of ignoring the user's learning effect of watching educational videos.

In terms of the impact of video quality on user experience, the results of this paper show that higher video quality significantly improves user learning effect, promotes positive emotional experience, and leads to more user engagement behaviors.

Video quality has a significant positive effect on user learning effect. Users can't learn video content without the quality of the video itself, when users watch the video to learn popular science content, the video's audio and picture are not synchronized, the layout is unreasonable, and other problems will make the user can't accurately understand the content that the video wants to convey, leading to the user's cognitive errors and misunderstandings of the popular science content; the video's blurred picture, poor sound quality, the misuse of special effects and other problems will hinder the user's learning of the content of popular science. The blurring of video images, poor sound quality, and abuse of special effects will hinder users' learning of popular science content, increase the difficulty of learning, and result in users' acquisition of video content failing to achieve the expected results. By maintaining better control over video quality, users can more accurately and smoothly grasp the information conveyed in the video, thereby improving the learning effect.

Video quality has a significant positive impact on user emotional experience. Relying on technical means such as high resolution, video encoding and streaming media, users with appropriate high-resolution display devices can watch clear online videos smoothly, improving the viewing experience and satisfaction. Therefore, in the current user-generated educational videos, 1080p, 2 k or even 4 k resolution videos have become the choice of most creators. When users watch educational videos with virtual avatars, high-resolution images are more in line with users' psychological expectations, allowing users to clearly access the content on the screen, and avoiding the negative emotions triggered by the image problem. At the same time, the virtual avatar as part of the video screen, the video display of the virtual avatar is clear and smooth, but also partly depends on the video quality itself, a clear picture helps to show the details of the virtual avatar cute or cool, to support the virtual avatar delicate and smooth movements and changes in the look, so that the user enjoys a better sense of view, which in turn produces positive emotional tendencies, favorites and sense of identity.

Video quality has a significant positive impact on user engagement. The popularity of high-definition videos and playback devices has gradually reduced users' tolerance for low-definition videos; at the same time, problems such as audio–video desynchronization and abusive special effects are further testing users' patience and bottom line. Therefore, all things being equal, the lower the quality of the video, the less attractive it is to users, the shorter the average viewing time, the fewer the comments, likes or favorites, and the less willing users are to share the video with their friends or on social media.

In conclusion, video quality has a significant positive effect on learning effect, emotional experience and user engagement. In order to improve user learning effect, induce good emotional experience and increase user engagement behavior, video creators in the production of learning videos containing virtual avatars should:

- Clear and smooth picture, should be used for users to watch, not less than 720p picture clarity, to ensure that the user can clearly see the details of the text and images in the video screen;
- To ensure that the quality of the audio to ensure that sound clear, no noise, moderate volume, and synchronized with the sound and picture, to avoid misunderstandings and cognitive difficulties caused by the mismatch between the sound and the image;
- reasonable layout, the layout of the video text, pictures, virtual avatars, subtitles and other elements should be reasonable, to avoid the virtual avatar on the blocking of the key information, and highlight important information and key points, to facilitate the user's rapid understanding;
- The appropriate use of special effects, too many of screen effects, virtual avatar effects, transitions and animation effects may distract the user's attention and reduce the learning effect, video creators should use these elements cautiously and only at the appropriate time in order to improve the user's emotional experience while ensuring the user's learning effect.

In terms of the impact of content quality on user experience, the results of this paper show that increased content quality reduces user learning effects, inhibits positive emotional experience, and decreases user engagement behaviors.

Content quality has a significant negative effect on user learning effect. Under the premise that the video content does not contain fallacies and is presented accurately, the higher the content quality, the higher its tier in Bloom's Cognitive Objective Taxonomy, and the more in-depth knowledge the video content involves and the more information and details it contains. Based on the cognitive load theory, video content that contains more information and details requires users to exert more attention, working memory, and cognitive effort to comprehend the material. This increased psychological burden can make the learning process more challenging^30^. In other words, for users with the same level of knowledge, they may encounter greater difficulties in watching videos with higher content quality, resulting in a poor learning effect.

Content quality has a significant negative impact on the emotional experience of users. Under the premise of ensuring basic quality, the improvement of content quality negatively affects user emotional experience through two aspects. On the one hand, when users find that the video content has a certain depth and amount of information, their viewing behavior starts to shift from pure entertainment to learning to a certain extent, which in turn reduces the level of relaxation, pleasure, and other emotions when watching videos. In other words, when watching learning science videos containing virtual avatars with higher content quality, users will be more serious. On the other hand, because the video content involves deeper knowledge and contains more information and details, users' cognitive load increases and the learning process becomes more difficult^30^. This further increases the possibility of negative emotions such as frustration and self-denial.

Content quality has a significant negative impact on user engagement. Similar to the effects on learning effect and emotional experience, as content quality increases, user engagement behavior is negatively affected, mainly in the form of a decrease in average viewing time, a decrease in sharing rate, and a greater reluctance of users to leave comments or interact with existing comments. This may be related to the fact that excessive content quality does not meet users' psychological expectations of videos. When users choose to watch a particular science video with a cute virtual avatar cover, they tend to form psychological expectations about the difficulty of the video content based on what they see on the cover, and the title of the video. During the viewing process, when users find that the difficulty of the content described in the video is beyond their learning level, they are very likely to stop the viewing behavior and choose to close the page or watch other videos. This directly leads to a decrease in the average viewing time and a decrease in the overall completion rate. At the same time, users who stop watching, close the page, or jump to another video page tend not to share the video or leave a comment.

In conclusion, content quality has a significant negative effect on learning effect, emotional experience and user engagement. In order to improve the learning effect, reduce the occurrence of negative emotions and promote user engagement, video creators should, on the premise of ensure accuracy and basic quality:

- Determine the depth and amount of information of the content according to the knowledge level and cognitive ability of the target users, avoiding overloading the cognitive ability of the users;
- Ensure a clear and concise hierarchical structure of the content, and provide appropriate guidance and sorting out in the process of conveying the knowledge, to Help users better understand and digest the knowledge.

In terms of the impact of virtual avatar expressiveness on user experience, the results of this paper show that high virtual avatar expressiveness can significantly improve user learning effect, lead to better emotional experience, and promote user engagement behavior.

Virtual avatar expressiveness has a significant positive effect on user learning effect. As a visual element of the screen, the significant positive effect of virtual avatar on learning effect is mainly realized by increasing users' interest in learning and enhancing emotional resonance and trust with users. Virtual avatars create strong visual attraction through exquisite appearance design and unique appearance features, so that videos containing virtual avatars stand out from many educational videos and arouse users' interest in learning. Users are more likely to have curiosity and motivation to understand the knowledge content of a beautiful and vivid virtual avatar. This attraction can motivate users to watch the video with continuous concentration and increase their motivation to learn^42^. By simulating the facial expressions and postures of real people and using real people's voices, virtual avatars are able to convey rich emotional information, shape their own personalities, enhance users' emotional resonance, and further help to improve the learning effect. In other words, as users' sense of "immersion" increases, they may learn more actively from the video content. The exquisite and delicate changes in the virtual avatar's expression and the natural and smooth changes in its movements are not only a reflection of the virtual avatar expressiveness, but also one of the ways to enhance the user's sense of trust. When the expressions and postures of virtual avatars look natural and vivid, users are more likely to develop a sense of fondness and trust for them, consider the knowledge information conveyed by the video reliable, and accept and understand the video content more easily.

Virtual avatar expressiveness has a significant positive impact on user emotional experience. The impact of virtual avatar expressiveness on user experience is mainly manifested in its ability to induce positive emotional tendencies, establish emotional ties with users, and enhance users' fondness and identification. Cute or cool virtual avatars can not only attract users' attention, but also stimulate users' interest and curiosity in the video, so that users are willing to actively click, watch and learn the video content, and ultimately induce positive emotional tendencies such as happiness, satisfaction or motivation. These virtual avatars can convey various emotional information, such as joy, surprise, regret, etc., in a variety of ways^5^, such as facial expressions and dubbed voice tones. These emotional expressions are an important way for virtual avatars to establish an emotional connection with users, which can stimulate user empathy and emotional engagement with the video content and enable users to feel the emotional experience and meaning more deeply when watching learning science videos.

Virtual avatar expressiveness has a significant positive effect on user engagement. For learning science videos containing virtual avatars, user engagement is reflected in indicators such as average viewing time, sharing rate, number of comments and activity. Virtual avatars with attractive appearance, behavior, and actions can continuously attract users' attention, and thus have a certain degree of effect on increasing users' average viewing time^31^. The emotional expressiveness of virtual avatars also has a positive impact on user engagement. The rich emotions and humanized qualities conveyed by virtual avatars can make it easier for users to establish emotional resonance with the avatars, increase their identification with and engagement in the video content, and make them more likely to love the virtual avatars in the video. Users will be more willing to watch and share videos with their favorite virtual avatars. Meanwhile, compared to ordinary Internet users, users tend to identify with and feel closer to other users who love the same virtual avatar, and are more willing to leave comments or engage in discussions. Sometimes, a group of virtual avatars may form spontaneously, and the communication and sharing among group members further increase the degree of user engagement in the videos.

In conclusion, virtual avatar expressiveness has a significant positive effect on learning effect, emotional experience and user engagement. Attractive virtual avatars can continue to attract users' attention, stimulate interest and more positive emotional experience, and promote the dissemination and sharing of videos. When creating educational videos containing virtual avatars, one should:

- Use visually appealing virtual avatars: combined with the preferences of the target users of the video, through the beautiful design of the stand-up drawings, it gives users a very cute or cool first impression, attracts their attention and stimulates their interest;
- Showing natural looks and movements: Exquisite and delicate looks and natural and smooth movements make the virtual avatar closer to the behavioral performance of real people in the user's cognition, so that the user is more likely to trust that the knowledge conveyed by the video is reliable, and is more likely to resonate with the virtual avatar and emotionally connect to it, and is more willing to continue to watch and interact with it;
- Convey feelings and personalities through virtual avatar: the ability to convey real feelings and personalities is the key to the emotional connection between users and virtual avatars and the key to the birth of favorite feelings, which is the "interesting soul" under the "good-looking skin". When users can feel the excitement, regret and other emotions of the virtual avatar, as well as humor, mystery and other personalities, they are more likely to gain a positive emotional experience, more likely to actively engage in the process of learning video content, and more likely to become a loyal fan of the virtual avatar.

## Conclusions

This paper constructs a conceptual model of user experience of educational videos containing virtual avatars, proposes research hypotheses, obtains user data through questionnaires, carries out reliability analysis, validity analysis, normality test and correlation analysis, and validates the research hypotheses using structural equation modeling. The results of the empirical study are as follows: a high level of video quality can significantly improve users' learning effect, emotional experience and user engagement; with the improvement of content quality, users' learning effect, emotional experience and user engagement level significantly decrease; a high level of virtual avatar expressiveness can significantly improve users' learning effect, emotional experience and user engagement level. In the creation of educational videos containing virtual avatars, creators should use visually appealing virtual avatars, show delicate and natural movements, convey feelings and personalities through virtual avatars, ensure clear video images and smooth audio, use reasonable scene and screen layouts, use special effects moderately, determine the depth of the video knowledge and the amount of information according to the knowledge level of the target user, and Demonstrate a clear content knowledge organization structure to improve the emotional experience of users before and after watching the video, and to improve the learning effect and user engagement after watching the video.

The shortcomings of the study and future research directions are as follows:

- The measurement of the learning effect of users in this study mainly adopts the indicators of learning interest degree and difficulty adaptation degree, and the data used are subjective evaluation data of users. Future research will use the controlled variable experimental method to collect objective score data by means of pre and post-tests to further verify the impact of the introduction of virtual avatars in educational videos on users' knowledge memorization and comprehension levels;
- The vast majority of the respondents of this study's questionnaire were Chinese undergraduate students aged 18–27 with undergraduate education and above, which does not have strong general applicability to social-facing online video users, and in the future, we can consider expanding the scope of users in the study.

## Data Availability

The dataset from the current analysis is not public but is available from the corresponding author upon reasonable request.
